# Large language models and the future of gastroenterology: dissecting the biopolitics of data in a global health ecosystem

**DOI:** 10.3389/fmed.2025.1668365

**Published:** 2025-08-20

**Authors:** Bilal Irfan, Roberto Sirvent

**Affiliations:** ^1^Center for Bioethics, Harvard Medical School, Boston, MA, United States; ^2^Center for Surgery and Public Health, Brigham & Women's Hospital, Boston, MA, United States; ^3^Department of Neurology, University of Michigan Medical School, Ann Arbor, MI, United States; ^4^Department of Epidemiology, University of Michigan School of Public Health, Ann Arbor, MI, United States; ^5^Department of Global Health and Social Medicine, Harvard Medical School, Boston, MA, United States

**Keywords:** global health, human dignity, artificial intelligence, large language model, biopolitics

## Introduction

Large language model (LLM) chatbots such as ChatGPT have moved from being a representation of technological novelty to working in clinical conversation in scarcely two years, fuelling hopes that conversational artificial intelligence will democratize expertise across gastroenterology and digestive endoscopy. A recent systematic review cataloged emerging LLM applications ranging from automated endoscopy report generation and guideline-concordant triage advice to interactive educational support for endoscopy teams (1). Emerging early evidence suggests that multimodal LLM frameworks now achieve near-expert accuracy in endoscopic lesion recognition while also producing plain-language explanations for patients, indicating that perhaps a single conversational platform could eventually unite diagnosis, structured documentation, and patient education (1–3). Yet the diffusion of any medical technology is shaped by social context, environmental externalities, and the epistemic boundaries of the data that train it. Taking stock of those wider forces is therefore essential if LLMs are to advance digestive health rather than reproduce existing inequities. By biopolitics we refer to the ensemble of policies, infrastructures, and market logics through which institutions manage bodies and populations, thus determining whose data are captured, which risks are rendered visible, and how care, surveillance, and environmental costs are distributed. Read through this lens, LLMs are not neutral engineering artifacts but instruments that channel attention and resources via training-data curation, platform governance, and procurement choices, with downstream consequences for equity in health outcomes.

## Clinical decision support at the scoping frontline

Endoscopists have long relied on pattern recognition; LLM-empowered computer vision now augments that skill with probabilistic reasoning that could perhaps flag subtle vascular patterns or borderline dysplasia that might otherwise elude the human eye. Beyond image interpretation, pilot work indicates that ChatGPT can automatically draft endoscopy reports and deliver guideline-aligned management advice, a combination that promises to lighten clerical workload and harmonize practice patterns across centers (1). Importantly, these gains are not confined to settings with a large amount of resources or available information. Some models may even maintain high accuracy even when queried with limited clinical context, a property that could help clinicians working in rural settings who lack subspecialty back-up. Nonetheless, hallucination remains a real hazard: observers have documented chatbots offering reassuring self-care for presentations that actually warrant urgent evaluation and presenting these suggestions with unwarranted confidence, illustrating how even a small fraction of erroneous outputs can jeopardize patient safety (4, 5). Rigorous human oversight and domain-specific fine-tuning therefore remain important safeguards to consider as decision support migrates from benchtop to bedside.

## Patient education and the moving frontier of evidence

The conversational ease of chatbots offers an antidote to the dense prose of some of the traditional patient leaflets, yet the value of any explanation rests on the scope and freshness of the underlying evidence. Environmental hazards illustrate the point. Recent umbrella and systematic reviews indicate that chronic exposure to fine particulate matter (PM_2_.5) is linked to higher colorectal-cancer incidence and mortality (roughly 18%−42% per 10 μgm^−^3) and to modest but consistent elevations in liver-injury enzymes, while evidence for inflammatory bowel disease (IBD) is suggestive yet drawn from heterogeneous individual studies rather than a formal meta-analysis, leaving considerable geographic and methodological variation unresolved (6, 7). No longer regarded as innocuous transit material, ingested micro- and nanoplastics are now shown to remodel intestinal microbial communities, erode the mucus-tight-junction barrier and amplify mucosal oxidative-inflammatory signaling, with experimental work in rodents, fish, chickens and human *in-vitro* gut models consistently reporting microplastic-driven dysbiosis, increased permeability, and exaggerated immune responses (8, 9). Unless LLMs continuously ingest such rapidly evolving literature, they risk giving patients obsolete reassurance or incomplete risk estimates. Retraining schedules, transparent citation of primary data, and clinician-controlled update mechanisms could be considered to be therefore baked into deployment plans from the outset.

It is worth noting that retrieval-augmented generation (RAG) can compel some models to ground responses in up-to-date bibliographic databases and guideline repositories, thereby returning linked citations and evidence grades at inference time. This approach can align with informatics guidance that search still matters in the generative era and with emerging ethics of AI-assisted evidence synthesis, including transparent provenance, librarian-curated query strategies using controlled vocabularies, and auditable update cadences (10–12).

## Occupational health, surveillance capital, and irritable bowel syndrome

The promise of just-in-time education must also be weighed against workplace realities. Irritable bowel syndrome (IBS) affects upwards of 10% of adults worldwide and is exquisitely sensitive to stress and restricted toilet access (13, 14). Qualitative interviews and occupational-health reviews show that many employees with IBS cope by rigidly controlling food intake, for example by bringing ‘safe' lunches, eating sparingly, or delaying meals, and by pushing through flares rather than taking sick leave, tactics adopted in response to tight schedules, limited bathroom access and high job demands; paradoxically, this hyper-vigilant self-management is associated with more severe symptoms, elevated presenteeism, and the kind of productivity losses that can curb advancement opportunities (15, 16). This tension is evident in Amazon's own fulfillment centers, where relentless rate-tracking and algorithmic surveillance are inseparable from workers' health: in UNI Global Union's 2023 *Life in the Amazon Panopticon* survey, a majority said the monitoring harmed their bodies and over half linked it to stomach pain, urgent toilet use, or other digestive distress, while a pending New Jersey lawsuit describes a warehouse associate with irritable-bowel syndrome who was terminated for “excessive” bathroom breaks after requesting medical accommodation (17, 18). Ironically, some of the same technology firms marketing “patient-friendly” chatbots are also exporting labor-disciplining algorithms. Gastroenterologists may therefore consider advocating not only for digital therapeutics but for the labor protections that enable patients to follow the advice, if accurate, coming from chatbots.

## Environmental and planetary health costs of conversational AI

Environmental exposure is another domain where LLM guidance could illuminate invisible risks. The experiences of Indigenous Yaqui communities in Sonora, Mexico offer some insights: organophosphate pesticides such as chlorpyrifos, methyl-parathion, and other chemicals that the United States has banned for use at home are still legally manufactured in U.S. plants and shipped to the Yaqui Valley under FIFRA and the Rotterdam Convention, a pattern that is a form of environmental violence (19). In a prospective cohort of more than 68,000 licensed pesticide applicators and their spouses, chronic use of several organochlorine and organophosphate pesticides, including dieldrin, toxaphene, parathion, and terbufos, was associated with significantly elevated hazards of incident inflammatory bowel disease, lending some epidemiologic support to existing experimental evidence that pesticide-induced gut dysbiosis and immune dysregulation can trigger IBD (20). Chatbots capable of translating toxicology data into timely warnings could help mitigate harm, yet such functionality also somewhat presupposes that registries from low- and middle-income settings are represented in the training corpus. Otherwise, the technology risks perpetuating what decolonial scholars call epistemicide, erasing non-Western evidence while centering agribusiness narratives.

Every prompt may also carry an unseen environmental invoice. Independent analyses indicate that a single training run of GPT-3 required an estimated 700,000 l of freshwater for data-center cooling, and Microsoft's own figures imply an annualized carbon footprint of about 8t CO_2_-e; researchers note that training such large models draws power on the order of gigawatt-hours (21). As automotive manufacturers embed voice assistants into petrol vehicles, the paradox deepens: the same model that advises patients on anti-reflux diets may indirectly contribute to particulate emissions implicated in esophageal adenocarcinoma. Mitigation strategies could include shifting computation to renewable-powered data centers, adopting parameter-efficient tuning rather than full retraining, and publishing life-cycle assessments with every major model release, though these might be contingent on what the evidence and health assessments display is feasible and warranted. Until such norms crystallize, gastroenterologists using chatbots must reckon with the possibility that clinical benefit for one patient could entail ecological harm for many others.

## Incarceration and the digital divide in chronic gastroenterology care

Carceral settings, including displaced people in detention facilities, can also serve to magnify barriers to digestive health (22). Data from the United States paints a consistent picture of structural disadvantage for people with IBD who are behind bars: a Massachusetts cohort found that patients who were incarcerated at the time of an IBD admission had almost four-fold higher odds of being readmitted to a hospital within a year than community patients (23). Similarly, a tertiary-center case series documents how imprisonment fragments every step of their care—biologic infusions are delayed for months or skipped altogether because transport cannot be arranged, nurse-led injection visits lapse, specialty-pharmacy refills falter, and even basic ostomy bags are in such short supply that leaks and skin breakdown occur, with little structured postoperative follow-up to address complications (24). Food insecurity is rising, affecting 13.5% of U.S. households in 2023, and among patients with inflammatory bowel disease it is linked to reduced diet quality, including higher intake of ultra-processed foods and greater risk of malnutrition (25, 26). While chatbots might theoretically deliver dietary counseling behind prison walls, real-world pilot projects show that even basic internet connectivity is restricted, and digital consultations are often monitored. Policymakers could therefore pair LLM rollouts with advocacy for guaranteed, confidential access and with reforms that address the underlying determinants of malnutrition.

## Discussion

The diversity of data found within LLM models also warrants greater attention. Here, diversity spans at least three strata. First is population diversity, that is demographic factors such as age, sex, ethnicity, language, geography, and socioeconomic position, so that minoritized populations and those from LMICs are not systematically under-represented. A second consideration is the diversity of the source and modality, for example non-English texts to ensure that knowledge is not narrowed to that is indexed in a few anglophone databases. There is also the need for methodological diversity in integrating different types of studies to ensure models can surface context-appropriate answers.

For example, *Moringa oleifera*, a drought-tolerant tree eaten widely from the Sahel to the Indian sub-continent, has demonstrated potent antioxidant activity and marked gastro-protection in rodent models, where leaf or polysaccharide extracts lowered ulcer indices, attenuated NF-κB-driven inflammation, preserved goblet cells, and maintained tight-junction proteins; these same preparations have also been shown to enrich beneficial taxa such as *Lactobacillus* in experimental studies, suggesting a capacity to modulate the gut environment, although confirmation in larger human cohorts is still pending (27, 28). Yet searches of leading LLM outputs may reveal scant mention of moringa when queried about dietary adjuncts for gastritis. Similar lack of mention of other information can affect other plant-based or traditional remedies, raising concern that proprietary training datasets privilege information indexed in large databases such as PubMed, or those readily accessible online. Open, multilingual, and peer-reviewed data pipelines are therefore essential if chatbots are to avoid reinforcing therapeutic monocultures.

Notwithstanding these early signals of utility, current LLM applications in gastroenterology may still face substantive limitations that provide a basis for an argument for some guard limits and a scoped deployment. Outputs remain somewhat non-deterministic and sensitive to prompt phrasing and hidden system instructions; fluency can mask fabrication of facts, rationales, or citations, and confidence may often be poorly calibrated to clinical risk. Many large language models provide limited provenance for the evidence they synthesize, and weight or policy updates complicate version control, reproducibility, and *post-hoc* audit. Privacy risks persist when protected health information is used for fine-tuning or inserted as in-context examples, with the possibility of memorization or leakage that institutional data-minimization and human-review policies do not uniformly address. Medico-legal responsibility for LLM-mediated advice and the environmental and opportunity costs of scaling also remain unsettled. These constraints suggest the utility of considering a supervised indication-specific use cases with clear off-ramps to human expertise, transparent provenance for cited evidence, rigorous external validation across sites and devices, and routine audits of calibration, drift, and equity prior to any expansion of scope.

LLM chatbots are poised to reshape gastroenterology, but their trajectory is neither linear nor value-neutral. At the clinical interface, they can sharpen optical diagnosis, streamline documentation, and translate guidelines into accessible language. In the public sphere, they could democratize knowledge about air pollution, pesticide exposure, and food insecurity, empowering communities that bear the brunt of digestive disease. These benefits, however, are arguably contingent on some five interlocking preconditions. First, continuous curation of training data could work to ensure timely incorporation of emerging evidence, from PM2.5 carcinogenicity to novel microbiome disruptors. Second, governance frameworks may need to demand transparent sourcing, rigorous human oversight, and algorithmic audit trails when applicable. Because hospital boards might be able to set strategy for the use of new technology, approve capital projects, and authorize vendor contracts, board composition will inevitably shape how and why LLM tools are procured and governed. Recent analyses show that many U.S. boards are dominated by finance and corporate executives with limited clinician or community representation, creating conflicts of interest that can tilt AI adoption toward factors other than patient safety, equity, and public-health commitments; AI oversight could therefore include board-level conflict disclosures and representative governance structures that center frontline clinicians and affected communities (29, 30). Third, equity mandates that analyze who, and at what cost, chatbots are accessible to people may be needed. Fourth, environmental accounting could accompany deployments, with carbon and water budgets published alongside accuracy metrics. Fifth, epistemic plurality requires deliberate inclusion of research from the Global South and from non-biomedical knowledge systems, lest LLM outputs perpetuate colonial hierarchies of evidence.

The gastroenterology community now stands at an inflection point. Clinicians, researchers, and patients have an opportunity to co-design conversational AI that advances digestive health while foregrounding labor rights, environmental stewardship, and epistemic justice. Realizing that vision will demand interdisciplinary collaboration that extends well beyond clinic walls, engaging computer scientists, ethicists, policy-makers, and, crucially, the communities most affected by digestive disease and digital inequity. If we succeed, the next generation of chatbots may be able to move beyond reciting guidelines, and actually work to help build a digestive health ecosystem that is sustainable, inclusive, and responsive to the complex realities of human biology and human society.
